# Multidimensional Profiles of Microbial Contamination and Hygiene Risk Across Functional Areas in Family Hotels

**DOI:** 10.3390/life16081278

**Published:** 2026-08-01

**Authors:** Alexander Martens, Markus Schauer, Susanne Mair, Mohamad Motevalli, Brigitte König

**Affiliations:** 1 Institute for Medical Microbiology and Virology, University of Leipzig Medical Center, 04103 Leipzig, Germany; 2 Department of Sport Science, German University of Health & Sport (DHGS), 85737 Ismaning, Germany; markus.schauer@dhgs-hochschule.de (M.S.); susanne.mair.dz@edu.dhgs-hochschule.de (S.M.); moha.motevalli@dhgs-hochschule.de (M.M.)

**Keywords:** environmental microbiology, microbial ecology, microbial communities, built environment, surface contamination, contact transmission, bacterial contamination, hygiene risk, culturable bacteria, infection prevention

## Abstract

Microbial contamination in hospitality settings remains understudied despite the high density of human contact and diverse operational activities that characterize hotel environments. This study aimed to characterize microbial contamination patterns and environmental hygiene risks across multiple functional areas of family-oriented hotels. A cross-sectional environmental microbiological investigation was conducted in family hotels in Bavaria, Germany. A total of 225 environmental surface samples were collected from guest rooms, child areas, food-related areas, service environments, and water-exposed locations. Bacterial isolates were identified using culture-based microbiology and MALDI-TOF mass spectrometry. Microbial prevalence, contamination severity, microbial richness, and pathogen prevalence were assessed using mixed-effects ordinal logistic regression, generalized additive model (GAM), and integrated hygiene profile, all performed in R (version 4.5.1). Microbial occurrence patterns differed markedly across functional areas. Contamination category distributions differed significantly among areas, with food-related environments showing the strongest enrichment in the highest contamination category (72.7%). Food-related areas showed significantly greater odds of severe contamination than guest rooms (OR = 8.37, 95% CI: 1.89–37.00), child areas (OR = 6.16, 95% CI: 1.20–31.46), and water-exposed environments (OR = 12.34, 95% CI: 2.45–62.10). Microbial richness differed across operational zones (*p* = 0.048) and was positively associated with contamination severity (β = 0.141, *p* < 0.001). GAM revealed a significant non-linear richness–contamination relationship (*p* < 0.001). Integrated hygiene profile consistently identified food-related and service areas as the highest risk environments. Environmental hygiene risks in hospitality settings display functional-area heterogeneity, highlighting the need for targeted, area-specific hygiene management strategies.

## 1. Introduction

The hospitality industry accommodates millions of travelers annually and represents a complex built environment characterized by intensive human contact, frequent turnover of occupants, diverse operational activities, and numerous shared surfaces [1,2,3]. Within hotels, guests interact with a wide range of environmental reservoirs, including guest-room furnishings, sanitary facilities, food-service areas, and recreational installations. These interfaces can facilitate the accumulation, persistence, and transmission of microorganisms originating from human, environmental, and operational sources [3,4,5,6]. Consequently, environmental hygiene has become an increasingly important component of quality management, infection prevention, and risk mitigation in hospitality settings [7,8].

Although healthcare-associated environmental contamination has been extensively investigated [9,10,11,12], substantially less attention has been directed toward hotel environments. Existing studies have demonstrated that frequently touched surfaces in accommodation facilities can harbor diverse bacterial communities, including opportunistic pathogens and hygiene-indicator microorganisms [3,4,13]. Moreover, studies of other high-occupancy built environments, including dormitories, cruise ships, commercial aircraft, and public accommodation settings, have shown that microbial contamination is influenced by human activity, environmental conditions, and hygiene practices [14,15,16,17]. The distribution of contamination is influenced by multiple factors, including occupant density, cleaning effectiveness, moisture exposure, food handling activities, and surface characteristics [2,3,4,18,19,20,21,22]. However, most available investigations have focused on limited sampling locations or individual contamination indicators, restricting a comprehensive understanding of contamination dynamics across operationally distinct hotel environments.

Multi-area indoor environments, including family-oriented hotels, constitute a highly relevant yet insufficiently studied setting. In addition to conventional guest accommodation, these facilities incorporate childcare units, recreational installations, wellness infrastructures, food-service operations, and service areas that differ substantially in human traffic, environmental conditions, and cleaning requirements [4,5,23,24]. Younger populations frequently interact with shared toys, play equipment, and other high-touch surfaces, making child-oriented environments particularly relevant settings for microbial transfer, accumulation, and environmental contamination [25,26,27]. Water-exposed environments may represent unique ecological niches where moisture availability, biofilm formation, and repeated guest exposure shape microbial persistence and contamination dynamics [28,29]. Food-related and service environments may create conditions favorable for microbial persistence and dissemination [30,31,32,33]. Collectively, these heterogeneous functional areas distinguish family hotels from conventional hotels by combining intensive child–adult interactions, moisture-associated recreational facilities, and diverse activity-specific environments within a single accommodation setting. Consequently, microbial contamination patterns in family hotels cannot be readily extrapolated from studies of standard hotels or other public indoor environments, highlighting the need for dedicated investigation. Therefore, understanding how contamination patterns differ across these functional areas of family hotels is essential for evidence-based hygiene management and targeted allocation of cleaning resources.

Beyond the mere detection of microorganisms, contemporary environmental hygiene assessment increasingly emphasizes multidimensional risk characterization [34,35]. Measures such as microbial richness, contamination burden, and pathogen prevalence provide complementary information regarding environmental microbial ecology and potential hygiene risk [36,37,38,39,40,41]. Thus, integrating these indicators may provide a more comprehensive assessment of environmental contamination than reliance on single microbiological endpoints alone. To our knowledge, no previous investigation has simultaneously evaluated contamination severity, microbial richness, and pathogen prevalence across operationally distinct functional areas within family hotels, leaving an important gap in the evidence base for risk-oriented environmental hygiene assessment.

The present study was designed to characterize microbial contamination patterns and environmental hygiene risks across multiple functional areas of family hotels in Bavaria, Germany. Through a structured environmental sampling approach combined with culture-based microbiology, MALDI-TOF mass spectrometric identification, and multivariate statistical analyses, we compared microbial prevalence, contamination severity, microbial richness, and pathogen occurrence across distinct operational zones of the hotel environment. Unlike previous hotel contamination surveys that primarily focused on selected surfaces or individual microbiological indicators, this study provides a comprehensive comparison of operationally distinct areas within family hotels by integrating multiple complementary measures of environmental contamination. In addition, we developed an integrated hygiene profile to identify areas associated with disproportionately elevated contamination burdens. By generating empirical evidence on area-specific microbial risks, this study contributes to the foundation for risk-based hygiene surveillance and targeted environmental management strategies within the hospitality sector.

## 2. Materials and Methods

### 2.1. Study Design and Setting

This study was conducted as a cross-sectional environmental microbiological investigation designed to characterize bacterial contamination patterns and environmental hygiene risks within family-oriented hotel facilities. Sampling was performed in two family hotels located in Bavaria, Germany. The investigation focused on a broad range of guest-accessible and operational environments with differing functions, occupant profiles, and expected contamination pressures. The study was conducted in accordance with applicable national regulations governing environmental microbiological investigations and institutional biosafety requirements. No human participants, biological specimens, or personal data were involved in the study.

The participating hotels represented large family-oriented accommodation facilities providing dedicated guest rooms, childcare services, wellness and spa areas, food-service facilities, and supporting operational units (kitchens, dishwashing facilities, and laundry rooms), thereby encompassing environments with diverse hygiene requirements and contamination pressures. Environmental sampling was conducted during multiple site visits in the summer season (July–August), corresponding to the peak operating period for family-oriented hotels. Sampling was scheduled to capture microbiological conditions across different functional areas under routine hotel operating conditions, with collections performed both following routine cleaning and during normal operation, depending on the operational characteristics of the sampled environment. Hotel occupancy rates and indoor environmental parameters (such as ambient temperature and relative humidity) were not systematically recorded during the sampling campaign because the primary objective was to assess microbiological contamination under routine operating conditions as part of environmental hygiene monitoring, rather than under controlled experimental conditions.

### 2.2. Environmental Sampling Strategy

A total of 225 environmental samples were collected from predefined sampling locations distributed throughout the hotels. Sampling locations were selected to capture microbiological conditions across areas differing in human traffic intensity, moisture exposure, food handling activities, and cleaning requirements. Sampled locations included guest rooms, childcare units (baby club, crawling club, mini club, adventure club, child area, and happy club), wellness facilities (frog tube, sauna, and family spa), buffet stations, kitchen workspaces, dishwashing facilities, laundry rooms and other operational service locations such as massage area, cosmetics unit, and birch rooms. Within each subarea, high-touch and hygiene-critical surfaces were systematically targeted. Sampled surfaces included sanitary fixtures (faucets, toilet handles, shower floors, soap dispensers, and cream dispensers), room-contact surfaces (light switches, door handles, telephones, television remote controls, and textiles), children’s toys and play equipment, spa and pool installations (railings, benches, slides, and paddling-pool components), as well as food-service and operational interfaces such as cutting boards, equipment control buttons, cleaned dishes, conveyor-belt surfaces, and laundry worktables.

Sampling was conducted following routine cleaning procedures and prior to guest or staff use whenever operationally feasible, thereby reflecting baseline post-cleaning environmental hygiene conditions. The study focused on residual bacterial contamination of environmental surfaces following standard cleaning procedures. For operational areas with continuous cleaning throughout the working day, including food-related and service areas, sampling was conducted during routine operations, reflecting standard operating conditions, as no discrete post-cleaning, pre-use interval was available. The hotels’ housekeeping programs included daily cleaning of guest rooms and sanitary facilities, while high-touch surfaces in public, childcare, wellness, and food-service areas were serviced at least daily or more frequently according to operational requirements. Housekeeping was performed by trained personnel using commercially approved cleaning and disinfection products in accordance with the hotels’ internal hygiene protocols and manufacturers’ instructions. No study-specific modifications to these procedures were implemented.

### 2.3. Sample Collection Procedures

Environmental samples were obtained using two standardized culture-based collection methods according to surface characteristics. Irregular, textured, or difficult-to-access surfaces were sampled using sterile ESwab^®^ collection systems containing liquid Amies transport medium. Flat and non-porous surfaces were sampled using contact (imprint) plates to obtain direct microbiological surface impressions. The choice of sampling method was based exclusively on the physical characteristics and accessibility of the sampled surface and was independent of the functional classification of the hotel area. Consequently, both sampling approaches were applied as appropriate to different surface types encountered throughout the investigated hotel environments. For ESwab sampling, the swab tip was first moistened in transport medium to enhance microbial recovery. The target surface was then sampled using a standardized multidirectional swabbing technique involving horizontal, vertical, and diagonal strokes under uniform pressure to maximize recovery from the entire accessible contact area. Following collection, swabs were immediately returned to transport medium and transported to the laboratory for processing. All samples were transported to the laboratory and processed within 24 h of collection.

### 2.4. Microbiological Culture and Isolation

A 10 µL portion of the ESwab transport medium was spread onto several culture media to improve recovery of a broad range of bacteria and to enable selective growth of clinically important species. The culture workflow used Müller-Hinton agar with 5% sheep blood to support broad bacterial growth, Mannitol salt agar to selectively recover staphylococci, and Endo agar to isolate and detect Gram-negative organisms. The ESwab transport medium was serially diluted (10-fold dilutions), and 10 µL aliquots from each dilution were inoculated onto each of the three culture media. For each dilution step, three plates of each culture medium were inoculated. Colony growth was evaluated according to the routine laboratory reporting procedure, including quantitative colony enumeration from the serial dilution cultures where applicable and semi-quantitative assessment of bacterial growth intensity. Colony counts were assessed on all three culture media, and the highest colony count for each microorganism was used for CFU determination. Samples collected using the contact (imprint) method were cultured exclusively on Tryptic Soy Agar (TSA) contact plates.

All inoculated media were incubated in an aerobic atmosphere (ambient air; without additional CO_2_ supplementation) at 37 °C in a laboratory incubator under standard humidified conditions and examined after approximately 24 h. Plates demonstrating limited or delayed growth were subjected to extended incubation for a total of 48–72 h and re-evaluation to allow detection of slower-growing organisms. Following incubation, colonies exhibiting distinct morphologies were selected for further characterization. Colony selection was based on visible differences in size, shape, pigmentation, texture, and hemolytic appearance where applicable. When multiple morphotypes were present within a sample, representative colonies from each morphotype were subcultured to obtain pure isolates and maximize recovery of within-sample microbial diversity.

### 2.5. Bacterial Identification

Pure isolates were identified using matrix-assisted laser desorption/ionization time-of-flight mass spectrometry (MALDI-TOF MS) with the VITEK MS system (bioMérieux, Lyon, France) using the commercial Knowledge Base v2.0 database for clinical use. Protein spectra were analyzed according to the manufacturer’s recommended procedures, and only identifications meeting the manufacturer’s acceptance criteria were accepted. Isolates that could not be identified by VITEK MS were subjected to 16S rRNA gene sequencing. Gram-negative isolates recovered on Endo agar were similarly identified using MALDI-TOF MS following purification by subculture. Presumptive *Staphylococcus aureus* isolates were additionally confirmed using a latex agglutination assay for coagulase detection. After identification, all confirmed isolates were preserved at −80 °C in cryoprotective storage medium for long-term storage and future phenotypic or molecular investigations.

Quality-control procedures were implemented throughout sample collection, culture, and identification stages. All culture media underwent routine sterility verification before use. Sample handling and microbiological processing were performed using aseptic techniques under standardized laboratory conditions. Negative-control procedures included periodic processing of unused swabs exposed to the sampling environment to monitor potential environmental or laboratory contamination. The performance and reliability of culture and identification workflows were monitored through routine internal quality-assurance procedures, including adherence to established laboratory quality-management principles and recognized culture-based microbiological standards [42,43].

### 2.6. Outcomes Variables

A sample was classified as microbiologically positive when at least one viable bacterial isolate was recovered and successfully identified following culture and laboratory analysis. Microbial richness was defined as the total number of distinct bacterial taxa identified within an individual sample. Richness was used as an indicator of within-sample microbial diversity and was analyzed as a count variable. Pathogen prevalence was calculated as the proportion of samples containing at least one bacterial taxon classified as clinically relevant or opportunistic pathogenic according to standard clinical microbiology references [44,45]. Only bacterial isolates identified at a taxonomic level sufficient for pathogenicity assignment were included. Genus-level identifications with insufficient species-level resolution and detected fungal taxa were not included in the pathogen prevalence calculation. The complete classification criteria are provided in Supplementary Table S1.

Microbiological burden was originally recorded using exact colony-forming unit (CFU) counts and/or standardized semi-quantitative laboratory reporting categories. For ESwab samples, CFU determinations were based on direct colony counts obtained following serial decimal dilution of the transport medium and culture on the appropriate microbiological media according to the routine laboratory protocol. Contact (imprint) samples were evaluated using TSA contact plates according to the routine laboratory microbiological assessment protocol. To facilitate standardized statistical analysis, each individual microbiological result was converted into a common five-level ordinal contamination severity variable before analysis. The conversion thresholds were not based on a specific published regulatory or microbiological standard, as no universally applicable CFU-based contamination threshold exists for the diverse sample types analyzed in this study. Instead, the five-level categorization was established as a study-specific ordinal classification to facilitate statistical analysis by reflecting increasing levels of bacterial burden. Accordingly, category 0 represented samples with no detectable bacterial growth, whereas category 1 corresponded to low contamination levels with reported values up to 50 CFU or equivalent low-level microbiological laboratory reporting categories. Category 2 denoted moderate contamination and included results corresponding approximately to the 50–10^2^ CFU range or equivalent intermediate laboratory reporting categories. Category 3 represented high contamination, encompassing values reported at approximately the 10^3^ CFU level. Category 4 reflected very high contamination and included samples reported at approximately the 10^5^ CFU level, those exceeding 10^5^ CFU, or samples exhibiting confluent growth.

Each sample, irrespective of whether it had been collected using an ESwab or a contact (imprint) plate, was assigned to the corresponding ordinal contamination category according to its original microbiological result. Because the two sampling methods differed in culture and enumeration procedures, statistical analyses were performed exclusively using the standardized ordinal contamination categories rather than directly comparing, combining, or averaging absolute CFU values obtained from the two methods. These ordinal categories were used exclusively as an analytical tool and should not be interpreted as universal microbiological contamination thresholds. This ordinal contamination scale served as the primary outcome measure of environmental contamination severity in all statistical analyses.

### 2.7. Functional Area Classification

For statistical analyses, individual sampling locations were aggregated into five predefined functional areas based on operational characteristics and expected contamination profiles: child area (*n* = 33), food-related area (*n* = 22), guest rooms (*n* = 93), service area (*n* = 26), and water-exposed area (*n* = 51). This classification scheme was established prior to analysis to improve interpretability, reduce sparsity among individual sampling locations, and facilitate comparisons of contamination patterns across major hotel functions.

### 2.8. Statistical Analysis

All statistical analyses and data visualizations were performed in R version 4.5.1 (R Foundation for Statistical Computing, Vienna, Austria). Microbial prevalence was calculated as the percentage of samples positive for each microorganism. Prevalence patterns and integrated hygiene profile indicators were visualized using heatmaps and hierarchical clustering based on Euclidean distance and Ward’s linkage method. Differences in contamination-category distributions among functional areas were assessed using chi-square tests of independence, with standardized Pearson residuals used to identify cells contributing to significant associations. Contamination severity was further evaluated using cumulative link mixed models (ordinal logistic regression), with contamination category as the ordered outcome, functional area as a fixed effect, and sampling subarea included as a random intercept to account for clustering of observations. Pairwise comparisons were expressed as odds ratios (ORs) with 95% confidence intervals (CIs). These comparisons were performed for descriptive/exploratory purposes, and *p*-values were not adjusted for multiple testing. Sensitivity analyses were performed using alternative reference categories. Microbial richness, defined as the number of microorganisms detected per sample, was compared among functional areas using the Kruskal–Wallis test. The association between richness and contamination severity was examined using Poisson regression. Potential non-linear relationships were further assessed using an ordinal generalized additive model (GAM) with an ordered categorical response distribution and microbial richness modeled as a smooth predictor of contamination category. Model-based predicted probabilities and 95% CIs were derived from the fitted GAM. An integrated hygiene profile was conducted by combining microbial richness, contamination severity (mean contamination-category score), and pathogen prevalence. Indicator values were standardized as z-scores and summarized using hierarchical clustering to identify similarities in environmental risk profiles among hotel functional areas. All tests were two-sided, and statistical significance was defined as *p* < 0.05.

## 3. Results

### 3.1. Microbial Prevalence Patterns

**Figure 1:**
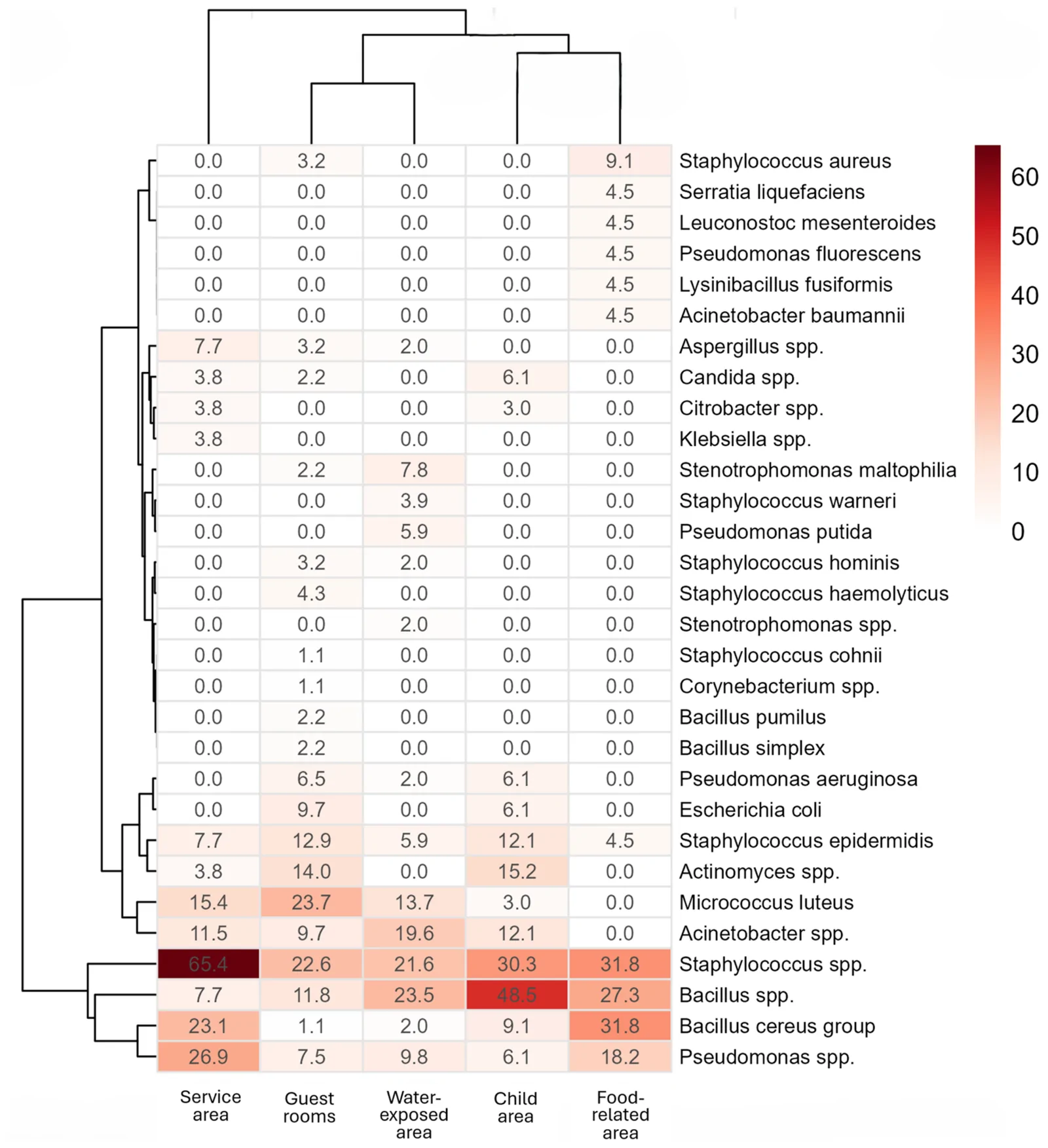
Distribution and clustering of microbial prevalence across hotel functional areas. Heatmap showing the prevalence (% positive samples) of detected microorganisms across five hotel functional areas. All detected taxa were included to provide a comprehensive overview of microbial occurrence patterns. Microorganisms were hierarchically clustered using Euclidean distance and Ward’s linkage to group taxa with similar prevalence profiles across hotel functional areas, facilitating visualization of shared occurrence patterns among microorganisms. Color intensity ranges from white (0%) to dark red (100%).

Microbial prevalence patterns differed across five functional areas (Figure 1). The predominant microorganisms identified were *Staphylococcus* spp. (65.4%) and *Pseudomonas* spp. (26.9%) in the service area; *Micrococcus luteus* (23.7%) and *Staphylococcus* spp. (22.6%) in guest rooms; *Bacillus* spp. (23.5%) and *Staphylococcus* spp. (21.6%) in water-exposed areas; *Bacillus* spp. (48.5%) and *Staphylococcus* spp. (30.3%) in the child area; and the *Bacillus cereus* group (31.8%) together with *Staphylococcus* spp. (31.8%) in the food-related area. Food-related and service areas generally displayed broader microbial occurrence profiles, while guest rooms were characterized by relatively lower prevalence for many detected taxa.

Hierarchical clustering grouped microorganisms according to similarities in their prevalence profiles across the five hotel functional areas. The analysis distinguished a cluster of dominant taxa, including *Staphylococcus* spp., *Bacillus* spp., the *Bacillus cereus* group, and *Pseudomonas* spp., which exhibited relatively high prevalence across multiple functional areas, from microorganisms detected only sporadically or largely restricted to individual areas. This analysis complemented the prevalence heatmap by providing an exploratory visualization of shared occurrence patterns among microorganisms, thereby facilitating comparison of overall distribution profiles rather than individual prevalence values alone.

### 3.2. Contamination Category Structure

**Figure 2:**
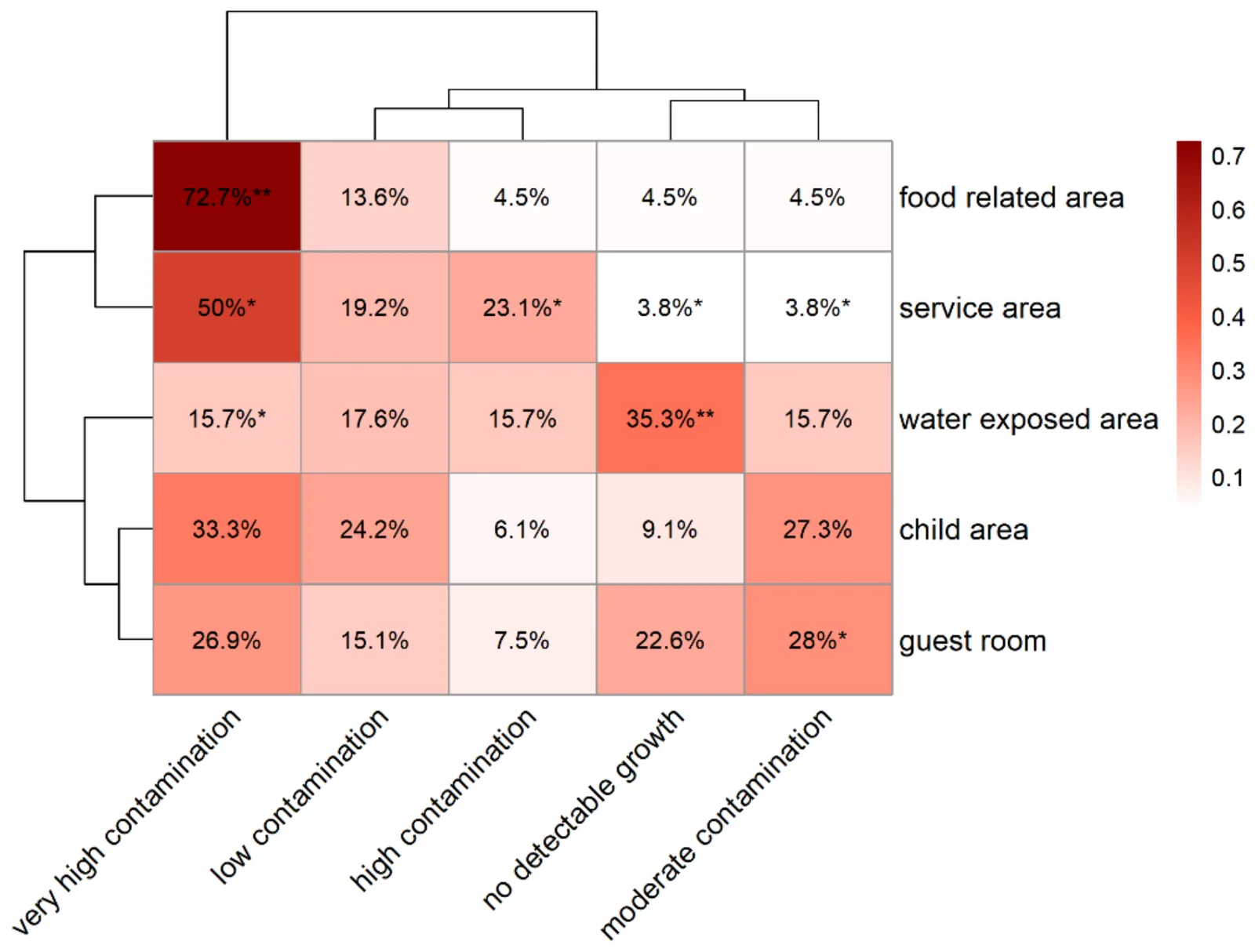
Distribution of contamination categories across hotel functional areas. Heatmap showing the observed proportion of samples within each functional area assigned to contamination categories 0–4. Color intensity ranges from white (0) to dark red (0.70). Asterisks denote significant standardized residuals from the chi-square test of independence (*|residual| > 1.96, *p* < 0.05; **|residual| > 3.0, *p* < 0.001). Rows and columns were hierarchically clustered using Euclidean distance and Ward’s linkage.

The distribution of contamination categories differed significantly among functional areas (Figure 2). The contamination heatmap demonstrated that food-related areas were strongly enriched in the highest contamination category (*p* < 0.001), with approximately 72.7% of samples classified as very highly contaminated. Service areas also showed a substantial concentration of samples within the highest contamination class (50.0%; *p* < 0.05), whereas water-exposed areas were characterized by a greater representation of no detectable growth. Guest rooms and child areas exhibited more heterogeneous contamination profiles, with observations distributed across multiple contamination categories. Hierarchical clustering showed that contamination severity was not randomly distributed throughout the hotel environment.

### 3.3. Contamination Severity Comparisons

Mixed-effects ordinal logistic regression analysis revealed several significant differences in contamination severity among functional areas (Table 1). Food-related areas exhibited significantly higher odds of belonging to a more severe contamination category than child areas (OR = 6.16, 95% CI: 1.20–31.46, *p* = 0.029), guest rooms (OR = 8.37, 95% CI: 1.89–37.00, *p* = 0.005), and water-exposed areas (OR = 12.34, 95% CI: 2.45–62.10, *p* = 0.002). Service areas also showed significantly greater contamination severity than water-exposed areas (OR = 4.69, 95% CI: 1.21–18.13, *p* = 0.025). A borderline association was observed between service areas and guest rooms (OR = 3.18, 95% CI: 0.96–10.49, *p* = 0.057), suggesting a tendency toward elevated contamination severity in service environments. Other pairwise comparisons were not statistically significant. Findings across alternative model parameterizations are presented in Supplementary Figure S1.

### 3.4. Microbial Richness

**Figure 3:**
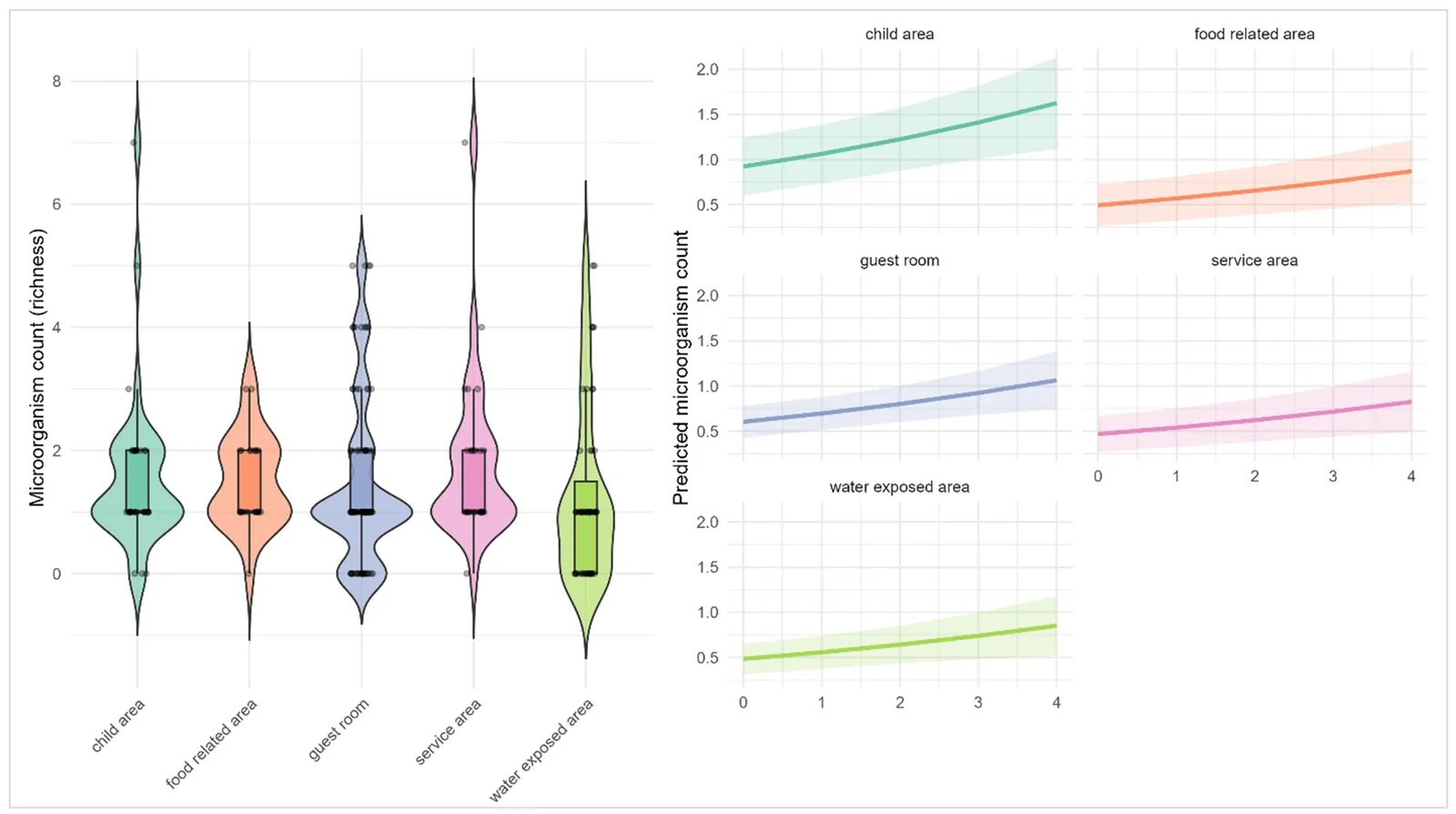
Microbial richness patterns and association with contamination severity. **Left panel**: Violin plots with embedded boxplots and individual observations showing the distribution of microbial richness across hotel functional areas. Kruskal–Wallis test showed a significant effect of area on microorganism counts (χ^2^ = 9.6, df = 4, *p* = 0.048). **Right panel**: Predicted microbial richness from a Poisson regression model with CFU category as the predictor. Shaded bands represent 95% confidence intervals. The estimated regression coefficient for CFU score was β = 0.141 (SE = 0.043; *p* < 0.001), indicating a significant positive association between contamination severity and microbial richness.

Microbial richness differed across hotel functional areas (Figure 3). The Kruskal–Wallis test revealed a significant overall effect of area on microorganism count (χ^2^ = 9.6, df = 4, *p* = 0.048). Child and service areas generally exhibited higher microorganism counts, whereas food-related and water-exposed areas displayed comparatively lower richness distributions despite their differing contamination profiles. Poisson regression analysis revealed a significant positive association between contamination severity and microbial richness (β = 0.141, SE = 0.043, *p* < 0.001). Predicted richness increased progressively across contamination categories, indicating that samples with greater microbial loads also tended to harbor more diverse microbial communities. This relationship was consistently observed across all areas, suggesting that contamination burden and microbial diversity are closely linked within hotel environments.

### 3.5. Richness-Contamination Relationship

**Figure 4:**
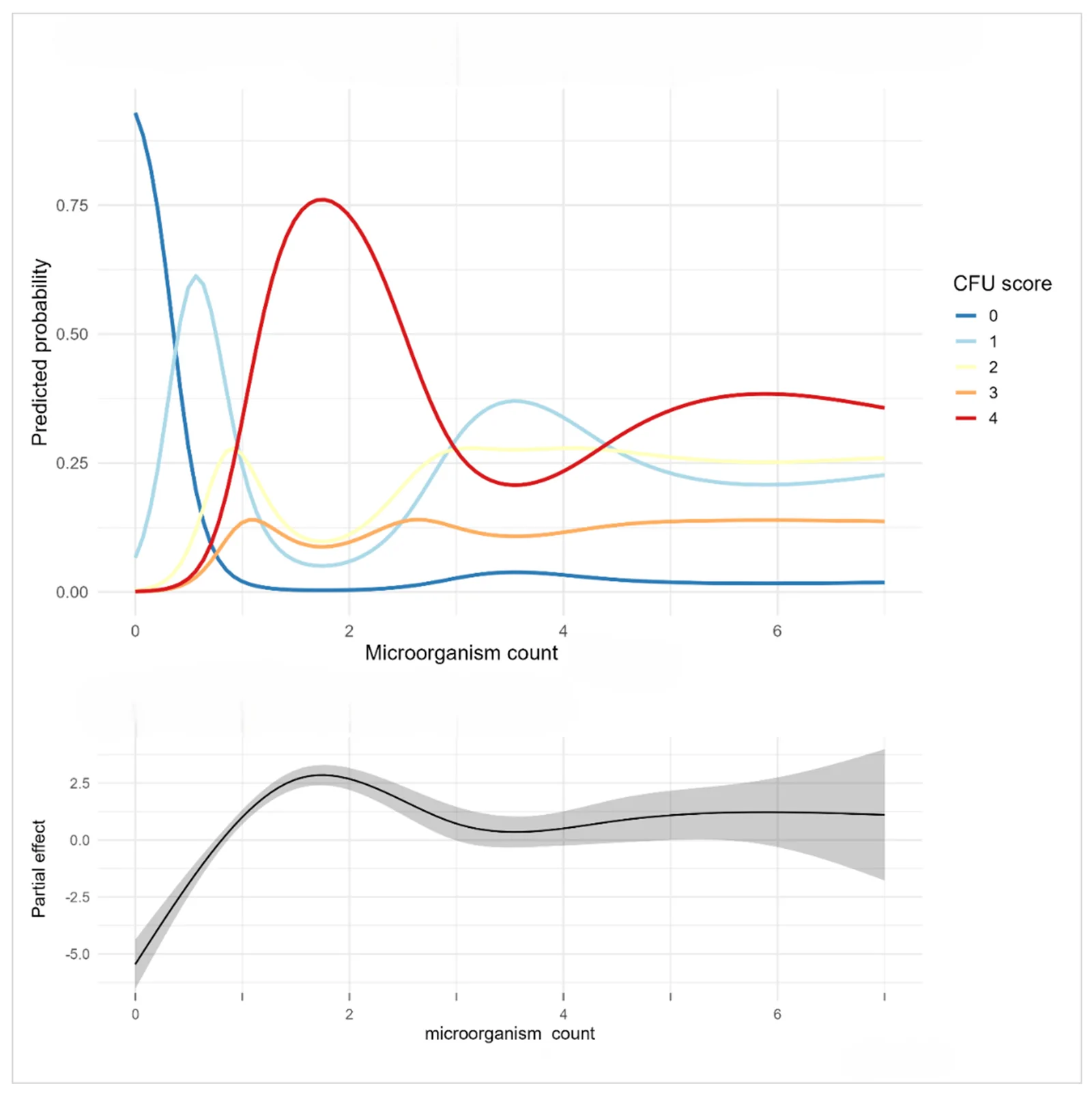
Non-linear association between microbial richness and contamination severity. Top panel: Predicted probabilities of contamination severity categories (CFU scores 0–4) as a function of microbial richness, estimated from an ordinal generalized additive model (GAM) using an ordered categorical response distribution (R = 5). Bottom panel: Smooth-term effect of microbial richness on the cumulative log-odds of higher contamination categories, shown with 95% confidence intervals. The smooth term was statistically significant (effective degrees of freedom, edf = 3.91; *p* < 0.001), indicating a non-linear relationship between richness and contamination severity.

The association between microbial richness and contamination severity was further evaluated using an ordinal GAM (Figure 4). The smooth term was significant (*p* < 0.001), indicating a pronounced non-linear association between richness and contamination risk. Predicted probabilities demonstrated that samples with low richness were most likely to belong to the lowest contamination categories, whereas increasing richness progressively shifted probabilities toward higher contamination classes. The shape of the smooth function suggested a threshold-like relationship in which early increases in richness were associated with substantial increases in contamination risk, followed by a more gradual increase at higher richness levels.

### 3.6. Integrated Hygiene Profile

**Figure 5:**
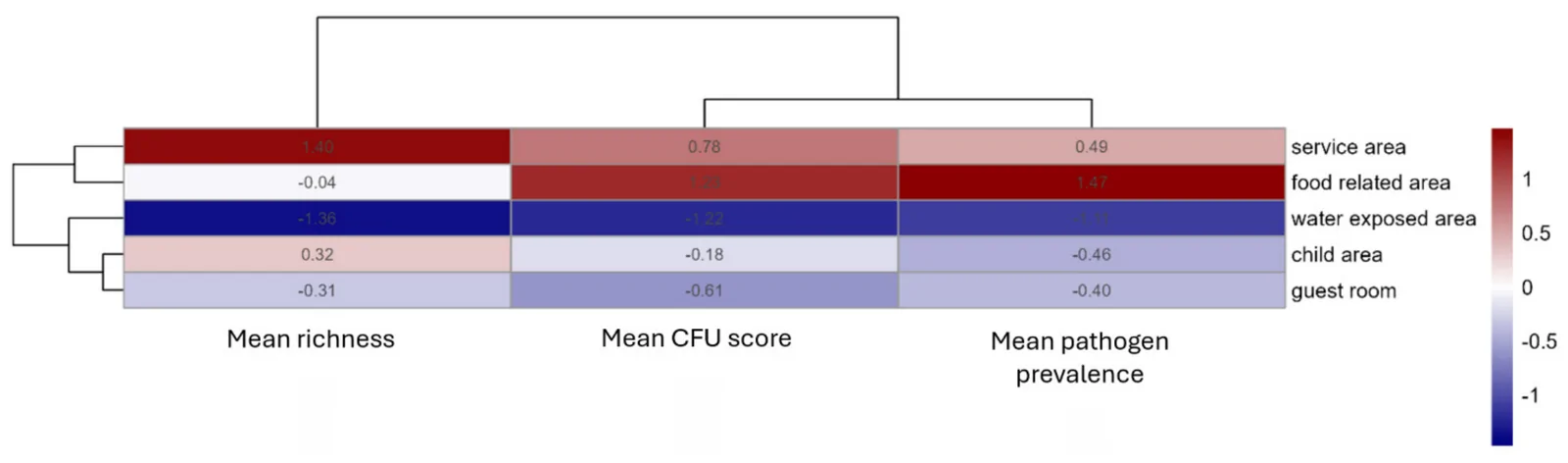
Integrated hygiene profile across hotel functional areas. Heatmap of standardized risk indicators, including mean microbial richness, mean CFU contamination score, and mean pathogen prevalence. Values are expressed as *z*-scores relative to the average. Positive values indicate above-average risk, while negative values indicate below-average risk. Rows and columns were hierarchically clustered using Euclidean distance and Ward’s linkage to identify similarities among environmental risk profiles.

To synthesize the multiple dimensions of hygiene profile, a composite risk assessment was performed using microbial richness, contamination severity, and pathogen prevalence (Figure 5). Across hotel functional areas, the integrated hygiene profile revealed marked heterogeneity in standardized risk indicators. Service areas showed consistently elevated values, with high mean microbial richness (z = 1.40), CFU contamination score (z = 0.78), and pathogen prevalence (z = 0.49), indicating an above-average composite risk. Food-related areas exhibited a distinct high-risk signature driven by microbial load and pathogens (mean CFU score z = 1.23, pathogen prevalence z = 1.47), despite near-average richness (z = −0.04). In contrast, water-exposed areas clustered as the lowest-risk environment, with uniformly negative z-scores for richness (z = −1.36), CFU contamination (z = −1.22), and pathogen prevalence (z = −1.11). Child areas showed slightly above-average richness (z = 0.32) but modestly reduced CFU (z = −0.18) and pathogen prevalence (z = −0.46), while guest rooms displayed a consistently below-average profile across all indicators (richness z = −0.31, CFU z = −0.61, pathogen prevalence z = −0.40). Hierarchical clustering of rows and columns highlighted the co-occurrence of high-risk signatures in service and food-related areas and a shared low-risk profile in water-exposed and guest room environments.

## 4. Discussion

This study provides a multidimensional assessment of microbial contamination dynamics across functionally distinct hotel environments, demonstrating that environmental hygiene risk is shaped by the operational characteristics of each area. The integrated interpretation of findings indicates that: (i) microbial prevalence patterns differed markedly across functional zones, with food-related and service areas exhibiting broader and more hygiene-relevant microbial profiles than guest rooms or water-exposed areas; (ii) contamination category distributions were highly uneven, with food-related areas showing the strongest enrichment in the highest contamination class (72.7% of samples classified as very highly contaminated) and service areas also displaying substantial clustering in severe categories; (iii) contamination severity comparisons revealed significantly higher odds of severe contamination in food-related and service areas relative to guest rooms, child areas, and water-exposed environments; (iv) microbial richness varied across functional areas and showed a robust positive association with contamination severity, supported by both linear and non-linear models; and (v) the composite risk assessment consistently identified food-related and service areas as the highest-risk environments, driven by elevated microbial loads, pathogen prevalence, and, in service areas, above-average richness.

A broader interpretation of the findings aligns with the built-environment microbiome framework, which holds that indoor microbial communities reflect combined inputs from human occupants, outdoor air and dust, water systems, food-handling materials, ventilation pathways, and building-surface characteristics [4,46,47]. A continental-scale microbiome study of hotel rooms showed that microbial richness and composition are shaped by environmental characteristics such as interior quality, floor type, and ventilation, with discordant patterns between richness and absolute quantity [17]. Our findings are consistent with this broader framework in showing that microbial richness and contamination burden can diverge across functional areas: food-related environments exhibited very high contamination severity but near-average richness, whereas service areas combined elevated richness, CFU burden, and pathogen prevalence. This pattern supports the notion that different operational zones within hotels host distinct microbial “niches” shaped by human activity, moisture, surface materials, and cleaning practices, in line with built-environment microbiome concepts that emphasize the combined influence of occupants, environmental inputs, and building characteristics on indoor microbial communities [4,19]. However, because the present study relied on culture-dependent methods, these observations should be interpreted as reflecting patterns within the viable and culturable fraction of the microbiota rather than the complete microbial community. Consequently, our findings provide indirect evidence of ecological differences among hotel environments but do not fully characterize microbial community structure or ecological interactions, which require culture-independent sequencing approaches [48]. Furthermore, although the number of samples analyzed was substantial, all samples were collected from only two family-oriented hotels in the same region; therefore, the observed contamination patterns should be interpreted as site-specific findings rather than representative of family hotels more broadly.

The predominance of staphylococci, bacilli, and other hygiene-relevant taxa across multiple areas aligns with previous work documenting frequent recovery of *Staphylococcus* spp. and other opportunistic bacteria from hotel rooms and university dormitories [3,13]. In particular, the high prevalence of staphylococci in guest rooms and service areas mirrors earlier observations that frequently touched surfaces (such as light switches, bathroom fixtures, and housekeeping equipment) can act as reservoirs for skin- and environment-associated bacteria in hospitality and residential settings [3,15,49,50]. At the same time, the relatively lower prevalence and risk indicators in guest rooms compared with food-related and service environments suggest that routine room-cleaning protocols may be more effective, or at least more consistently implemented, than hygiene practices in operational back-of-house areas.

A functional interpretation of the risk landscape highlights distinct microbiological roles for each hotel area. The consistently elevated contamination burden in food-related environments is congruent with evidence from food-processing and food-service settings, where food-contact and adjacent non-food-contact surfaces frequently harbor persistent communities dominated by genera such as *Staphylococcus*, *Pseudomonas*, *Acinetobacter*, and *Bacillus cereus*, supported by nutrient availability, moisture, and repeated microbial introduction that favor biofilm formation and recurrent contamination [30,33]. Although the differing sampling conditions across functional areas should be considered when interpreting these comparisons, the observed pattern remains biologically plausible given the operational characteristics of these environments. The ability of food-associated bacteria to form biofilms on equipment and food-contact surfaces has been repeatedly linked to recurring contamination and reduced efficacy of conventional cleaning and sanitizing procedures [51,52]. These parallels reinforce the biological plausibility of the higher contamination levels observed in hotel food-service zones under the sampling conditions applied in this study and support their consideration as priority targets for risk-based hygiene surveillance and preventive interventions within the investigated hotels. Nevertheless, because sampling conditions differed between functional areas, these findings should not be interpreted as demonstrating that food-service environments are inherently higher-risk than other hotel settings. Service areas, although less visible to guests, emerged as additional high-risk nodes, which is consistent with studies showing that microorganisms accumulate in environments characterized by repeated human movement, equipment handling, and material transfer, and that dispersal and repeated microbial introduction are key determinants of community assembly in built environments [4,19], with occupant movement and recirculated air driving microbial build-up in HVAC filters and other operational reservoirs [41,53,54]. Given their connective function between guest rooms, food-service operations, childcare facilities, and external inputs, service areas may act as hubs for microbial redistribution and therefore warrant explicit inclusion in targeted hygiene strategies.

Child-oriented areas displayed only moderate contamination levels but remain microbiologically important because of the distinct exposure pathways and susceptibilities of children. Higher hand-to-mouth activity, frequent contact with shared objects, immature immune function, and close interpersonal interactions can amplify the consequences of even modest environmental contamination [26,27,55]. Studies in childcare facilities further indicate that occupant density and usage patterns strongly shape microbial community composition, with high-contact surfaces serving as key reservoirs of human-associated microorganisms [25]. Accordingly, risk assessment in child-focused hotel environments should integrate both contamination burden and child-specific behavioral exposure patterns. By contrast, the relatively favorable risk profile of water-exposed environments in this study, despite the recognized role of moisture in supporting microbial survival, aligns with observations that wet indoor niches (e.g., drains, showerheads, plumbing) often harbor specialized, biofilm-forming communities with lower taxonomic diversity than dry surfaces, where microbes accumulate from multiple sources over time [28,56,57,58]. In water-related indoor biomes, including domestic appliances and plumbing systems, these specialized communities are frequently dominated by stress-resistant, sometimes opportunistic taxa, reflecting strong selection by moisture, temperature, and detergent regimes rather than high overall richness [28,57].

The strong enrichment of very high contamination categories in food-related and service areas, and the substantially higher odds of severe contamination in these zones compared with guest rooms, child areas, and water-exposed environments, are consistent with the broader literature on fomite-mediated risk in high-contact, operational settings. Evidence has shown that many clinically relevant bacteria and fungi can remain viable for weeks to months on dry surfaces, with survival strongly modulated by surface material, temperature, and humidity [2]. These characteristics are typical of food-service and service environments, where repeated handling, intermittent moisture, and complex surface topographies may favor both deposition and persistence of microorganisms [59,60].

Our findings also resonate with experimental work in a hotel lobby, where a viral tracer seeded on a small number of high-touch surfaces spread to 50% of sampled fomites within 4 h, and a risk-based targeted hygiene intervention achieved a ~97% reduction in viral concentrations and infection risk [5]. The concentration of high contamination categories in food-related and service areas in the present study suggests that similar targeted, risk-based hygiene strategies (prioritizing high-touch, high-load surfaces and critical time points) could be particularly impactful in family hotels. This is further supported by quantitative microbial risk assessment (QMRA) evidence showing that initial surface concentrations and surface-to-hand transfer efficiencies are key determinants of infection risk [5], highlighting the importance of reducing contamination burden at its primary environmental sources.

The positive association between culture-derived microbial richness and contamination severity, observed both in Poisson regression and in the ordinal generalized additive model, indicates that samples with higher CFU burdens also tended to harbor a greater number of cultured taxa. The GAM results suggested a threshold-like relationship, with early increases in richness associated with steep increases in contamination risk, followed by a more gradual rise at higher richness levels. This pattern is compatible with ecological interpretations in which low-richness states reflect relatively clean or recently sanitized surfaces, whereas intermediate richness may mark the transition to more established, multi-source microbial assemblages with higher overall biomass [61,62,63]. However, because both contamination severity and culture-derived microbial richness were derived from the same culture-based samples, part of this association may also reflect the greater likelihood of recovering and identifying additional colony morphotypes from samples with higher bacterial loads, rather than solely an underlying ecological relationship. Accordingly, the observed association should be interpreted as reflecting both biological and methodological influences.

In the broader built-environment literature, higher diversity on dry surfaces (e.g., dust, HVAC filters) compared with wet niches has been reported, with dry reservoirs accumulating diverse airborne and occupant-derived microbes over time, whereas wet niches such as drains, showerheads, and water-using appliances are dominated by specialized, biofilm-forming taxa adapted to high moisture and selective chemical environments [19,28,64,65]. The present study, which focused on culturable bacteria and surface CFU categories, cannot fully resolve these ecological nuances, but the observed richness–severity relationship suggests that, within the culturable fraction, diversity may serve as a proxy for cumulative contamination pressure and multi-source microbial input. At the same time, culture-based methods capture only a subset of the total microbiome, and the “great plate count anomaly” implies that true diversity is substantially underestimated [4]. Thus, the richness-risk relationship documented here likely reflects a conservative estimate of underlying ecological complexity.

By combining microbial richness, contamination severity, and pathogen prevalence into a standardized composite profile, this study delineates a clear risk landscape across hotel functional areas. Service and food-related environments clustered as high-risk zones, whereas water-exposed areas and guest rooms formed a low-risk cluster, with child areas occupying an intermediate position. This pattern aligns with conceptual and empirical work indicating that indoor microbial contamination is driven by the interplay of human occupancy, surface type, moisture, and operational practices [4,19,41]. It also complements evidence from dormitory and campus studies showing that surface contamination can be widespread but not uniformly associated with reported illness, with some cohorts demonstrating substantial bacterial burdens on high-touch surfaces (e.g., computer keyboards, telephones, elevator buttons) without clear correlations to self-reported health outcomes [13,49,50]. Importantly, the present findings support a shift from uniform, area-agnostic cleaning protocols toward functionally differentiated, risk-based hygiene management in family hotels. Evidence from hotel lobbies and other communal environments indicates that targeted interventions focusing on high-touch, high-risk surfaces can substantially reduce contamination and modeled infection risk while avoiding excessive chemical use and “hygiene theater” [5]. Our integrated hygiene profile provides an empirical basis for prioritizing food-related and service environments as primary targets for intensified cleaning, monitoring, and staff training, while maintaining appropriate but potentially less intensive protocols in lower-risk zones such as guest rooms and water-exposed areas.

Several limitations should also be considered. First, sampling was restricted to two family-oriented hotels located within a single geographic region, and although the sampling coverage within these settings was extensive, the limited number of participating facilities restricts the generalizability of the findings. Therefore, the results should be considered descriptive of the studied hotel environments and should not be extrapolated to all family-oriented hotels or other hospitality settings without further validation across a wider range of facilities. Second, hotel occupancy rates and indoor environmental parameters (ambient temperature and relative humidity) were not systematically recorded. As sampling was performed under routine operating conditions, the potential influence of these factors on bacterial recovery could not be quantitatively assessed. In addition, the cross-sectional design captures only a single temporal snapshot and cannot account for seasonal variability, fluctuations in occupancy, or changes in operational practices. In addition, formal assessment of the proportional-odds assumption for the ordinal models and overdispersion diagnostics for the Poisson model was not performed; therefore, these analyses should be interpreted primarily as exploratory assessments of associations within the sampled dataset. Another limitation is that sampling was not performed under identical operational conditions across all functional areas. While most areas were sampled following routine cleaning, food-related and service areas were sampled during routine operations due to the absence of a discrete post-cleaning interval, which should be considered when interpreting comparisons between functional areas. An additional limitation is that two different surface sampling methods (ESwab and contact plates) were used according to surface characteristics; therefore, differences in microbial recovery efficiency between the methods should be considered when interpreting comparisons across functional areas. Moreover, the culture-based approach was designed to detect viable microorganisms relevant to hygiene assessment and therefore does not capture the full diversity of environmental microbiota. Culture-dependent methods recover only a subset of environmental microbial communities, and ecological interpretations are therefore limited to the culturable fraction. Although MALDI-TOF MS provided high-resolution identification of cultured isolates, culture-independent approaches such as 16S rRNA gene sequencing and shotgun metagenomics would enable a more comprehensive characterization of microbial diversity, community structure, and functional potential. Nevertheless, the recovered isolates included both common human-associated and diverse environmental microorganisms, suggesting that the major microbial groups present in the sampled environments were adequately represented.

Despite these limitations, the study has several strengths. The investigation included a diverse range of environmental settings encompassing guest rooms, child-oriented areas, food-service facilities, service environments, and water-exposed locations. Standardized culture-based microbiological procedures were combined with MALDI-TOF mass spectrometry, a highly reliable and increasingly adopted technique for rapid microbial identification in indoor environmental studies [4]. The application of mixed-effects modeling, generalized additive modeling, and integrated hygiene profile provided complementary analytical perspectives and enabled a comprehensive evaluation of contamination dynamics across hotel environments. Furthermore, the incorporation of microbial richness, contamination severity, and pathogen prevalence into a unified framework represents a novel contribution to environmental hygiene assessment in hospitality settings.

Future studies should therefore incorporate longitudinal sampling designs, larger numbers of hotels across diverse geographic regions and operational settings, culture-independent sequencing approaches (e.g., 16S rRNA gene sequencing and shotgun metagenomics), and assessments of antimicrobial resistance and biofilm-associated persistence. Integrating environmental microbiology with occupancy data, cleaning practices, ventilation characteristics, and building design variables would further improve understanding of the mechanisms driving contamination dynamics in hospitality environments and help determine whether the observed patterns are generalizable across the broader hotel sector.

## 5. Conclusions

The present study provides a comprehensive assessment of microbial contamination patterns across functionally distinct environments within the two investigated family-oriented hotels and demonstrates that environmental hygiene risks are not uniformly distributed throughout hospitality settings. The findings revealed a consistent pattern of higher contamination in food-related and service areas than in guest rooms and water-exposed locations; however, these comparisons should be interpreted in the context of the differing sampling conditions across functional areas. These results highlight the importance of considering the operational function of hotel spaces when evaluating environmental hygiene and microbial exposure risks. The observed association between microbial richness and contamination severity further suggests that ecological indicators may offer valuable complementary information for environmental risk assessment beyond conventional contamination measures alone. Collectively, the findings support the concept that microbial contamination in hotels is shaped by the interaction of human activity, environmental conditions, surface characteristics, and operational practices, consistent with contemporary built-environment microbiome frameworks. However, given the limited number of participating hotels, these findings should be interpreted as site-specific observations requiring validation in broader hospitality settings.

From a practical perspective, the study provides empirical evidence supporting the implementation of risk-based hygiene management strategies that prioritize monitoring, cleaning, and staff training in food- and service-related environments, where the highest contamination levels were observed under the sampling conditions used in this study, rather than relying solely on uniform cleaning protocols across all hotel areas. Such targeted approaches may improve hygiene outcomes, optimize resource allocation, and contribute to safer hospitality environments for both guests and staff.

## Figures and Tables

**Table 1:** Pairwise comparisons of contamination severity among hotel functional areas based on mixed-effects ordinal logistic regression.

Pairwise Area Comparisons	Child Area	Food-Related Area	Guest Rooms	Service Area	Water-Exposed Area
**Child area**	-	6.16	1.36	0.43	2.00
		(1.20–31.46)	(0.45–4.13)	(0.11–1.68)	(0.56–7.21)
		*p* = 0.029	*p* = 0.589	*p* = 0.224	*p* = 0.287
**Food-related area**	0.16	-	8.37	2.63	12.34
	(0.03–0.83)		(1.89–37.00)	(0.49–14.06)	(2.45–62.10)
	*p* = 0.029		*p* = 0.005	*p* = 0.258	*p* = 0.002
**Guest room**	0.74	0.12	-	3.18	1.47
	(0.24–2.24)	(0.03–0.53)		(0.96–10.49)	(0.50–4.37)
	*p* = 0.589	*p* = 0.005		*p* = 0.057	*p* = 0.484
**Service area**	2.34	0.38	0.31	-	4.69
	(0.59–9.21)	(0.07–2.03)	(0.10–1.04)		(1.21–18.13)
	*p* = 0.224	*p* = 0.258	*p* = 0.057		*p* = 0.025
**Water-exposed area**	0.50	0.08	0.68	0.21	-
	(0.14–1.79)	(0.02–0.41)	(0.23–2.01)	(0.06–0.82)	
	*p* = 0.287	*p* = 0.002	*p* = 0.484	*p* = 0.025	

Note: Odds ratios (ORs) represent the relative odds of belonging to a higher CFU contamination category for the row area compared with the column reference area. Models included a random intercept for sampling subarea to account for clustering of repeated observations (27 subareas; n = 225 samples). Confidence intervals are 95% Wald confidence intervals. Statistical significance was assessed using Wald tests. Model fit statistics were consistent across specifications (McFadden’s pseudo-R^2^ = 0.0174; AIC = 668.8).

## Data Availability

The data supporting the findings of this study are available from the corresponding author upon reasonable request.

## Supplementary Materials

The following supporting information can be downloaded at https://www.mdpi.com/article/10.3390/life16081278/s1. Figure S1: Sensitivity analysis of contamination-risk comparisons using alternative reference categories. Table S1: Classification criteria for bacterial taxa considered clinically relevant or opportunistically pathogenic in the pathogen prevalence analysis.

## Abbreviations

The following abbreviations are used in this manuscript:

MALDI-TOF	Matrix-Assisted Laser Desorption/Ionization Time-of-Flight
CFU	Colony-Forming Unit
OR	Odds Ratio
CI	Confidence Interval
df	Degrees of Freedom
edf	Effective Degrees of Freedom
SE	Standard Error
HVAC	Heating, Ventilation, and Air Conditioning
QMRA	Quantitative Microbial Risk Assessment
TSA	Tryptic Soy Agar
