# Supplementary material for: Multidimensional Profiles of Microbial Contamination and Hygiene Risk Across Functional Areas in Family Hotels
Source: Life (Basel). 2026 Aug 1;16(8):1278. doi: 10.3390/life16081278 (PMC13514714; doi:10.3390/life16081278)
Supplement: Supplementary file 1 [file life-16-01278-s001.zip › life-4438650-supplementary.pdf]

Supplementary Materials

Table S1. Classification criteria for bacterial taxa considered clinically relevant or opportunistically pathogenic in the pathogen prevalence analysis

Identified taxon	Included in analysis	Classification category	Rationale for classification
Staphylococcus aureus	Yes	Established pathogen	Major human pathogen associated with skin and soft tissue infections, bloodstream infections, and foodborne disease
Staphylococcus epidermidis	Yes	Opportunistic pathogen	Clinically relevant coagulase-negative staphylococcus associated with device-related and bloodstream infections
Staphylococcus hominis	Yes	Opportunistic pathogen	Coagulase-negative staphylococcus associated with opportunistic bloodstream infections
Staphylococcus haemolyticus	Yes	Opportunistic pathogen	Clinically relevant coagulase-negative staphylococcus associated with healthcare-associated infections
Staphylococcus warneri	Yes	Opportunistic pathogen	Reported as an opportunistic cause of invasive infections
Staphylococcus cohnii	Yes	Opportunistic pathogen	Rare but documented opportunistic human infections
Staphylococcus spp.	No	Not included	Genus-level identification does not allow reliable assignment of pathogenic potential
Micrococcus luteus	No	Environmental /commensal organism	Predominantly associated with normal skin flora and environmental habitats; limited pathogenic relevance
Bacillus cereus group	Yes	Environmental opportunistic pathogen	Associated with foodborne illness through toxin production and occasional invasive infections
Bacillus pumilus	No	Environmental organism	Limited evidence of human pathogenicity; mainly environmental
Bacillus simplex	No	Environmental organism	No established clinical relevance
Bacillus spp.	No	Not included	Species-level identification insufficient for pathogenicity classification
Lysinibacillus fusiformis	No	Environmental organism	Rare clinical relevance and insufficient evidence for routine pathogenic classification
Actinomyces spp.	No	Commensal /opportunistic genus	Many species represent endogenous flora; pathogenicity depends on species and clinical context
Acinetobacter baumannii	Yes	Opportunistic pathogen	Important healthcare-associated pathogen with recognized antimicrobial resistance relevance
Acinetobacter spp.	No	Not included	Genus-level identification insufficient because pathogenic potential varies among species

Pseudomonas aeruginosa	Yes	Opportunistic pathogen	Environmental organism with well-established association with human infections
Pseudomonas putida	No	Environmental organism	Primarily environmental species with rare opportunistic infections
Pseudomonas fluorescens	No	Environmental organism	Primarily environmental organism with limited clinical relevance
Pseudomonas spp.	No	Not included	Species-level identification insufficient for pathogenicity assignment
Stenotrophomonas maltophilia	Yes	Opportunistic pathogen	Clinically relevant opportunistic pathogen associated with infections in vulnerable individuals
Stenotrophomonas spp.	No	Not included	Species-level identification insufficient for reliable classification
Escherichia coli	Yes	Established pathogen	Important human pathogen associated with intestinal and extraintestinal infections
Citrobacter spp.	Yes	Opportunistic pathogen	Enterobacterales genus associated with opportunistic infections
Klebsiella spp.	Yes	Opportunistic pathogen	Clinically relevant Enterobacterales genus associated with healthcare-associated infections
Leuconostoc mesenteroides	No	Food-associated organism	Mainly food-associated organism with limited pathogenic relevance
Serratia liquefaciens	Yes	Opportunistic pathogen	Reported in healthcare-associated and opportunistic infections
Candida spp.	No	Not included	Fungal organisms were outside the bacterial pathogen prevalence analysis
Aspergillus spp.	No	Not included	Fungal organisms were outside the bacterial pathogen prevalence analysis
Corynebacterium spp.	No	Commensal /opportunistic genus	Predominantly normal skin flora; pathogenicity depends on species-level identification

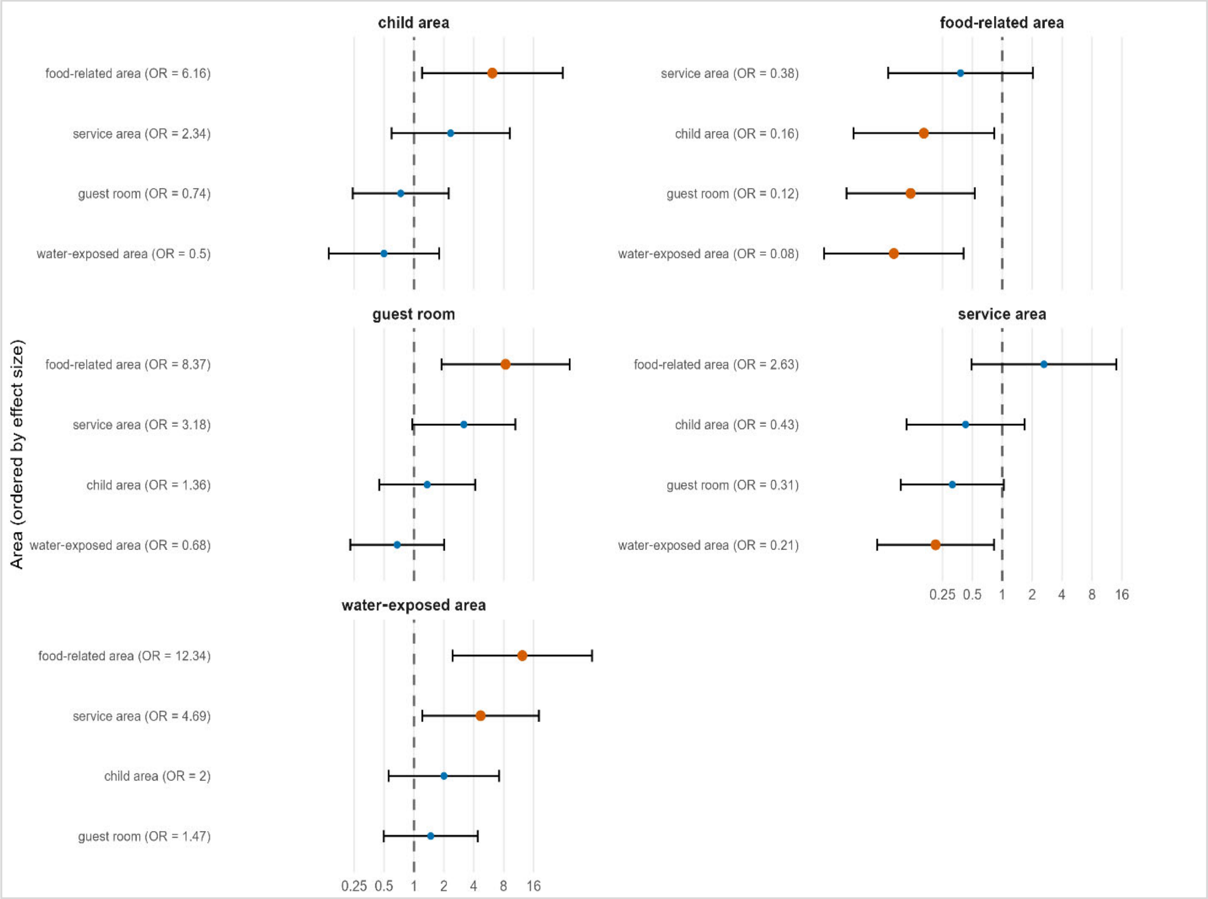

Figure S1. Sensitivity analysis of contamination-risk comparisons using alternative reference categories. Forest plots showing odds ratios (ORs) from cumulative link mixed models with a random intercept for subarea. Each panel represents a separate model using a different functional area as the reference category. Error bars represent 95% confidence intervals displayed on a logarithmic scale. Orange symbols indicate statistically significant comparisons ($p < 0.05$), whereas blue symbols indicate non-significant comparisons.
